# Compliance with recommendations limiting COVID-19 contagion among university students in Sweden: associations with self-reported symptoms, mental health and academic self-efficacy

**DOI:** 10.1177/14034948211027824

**Published:** 2021-07-02

**Authors:** Anne H. Berman, Marcus Bendtsen, Olof Molander, Petra Lindfors, Philip Lindner, Lilian Granlund, Naira Topooco, Karin Engström, Claes Andersson

**Affiliations:** 1 Department of Psychology, Uppsala University, Sweden; 2 Centre for Psychiatry Research, Karolinska Institutet and Stockholm Health Care Services, Sweden; 3 Department of Health, Medicine and Caring Sciences, Linköping University, Sweden; 4 Department of Psychology, Stockholm University, Sweden; 5 Department of Behavioural Sciences and Learning, Linköping University, Sweden; 6 Department of Global Public Health, Karolinska Institutet, Sweden; 7 Department of Criminology, Malmö University, Sweden

**Keywords:** COVID-19, pandemics, epidemiology, social medicine, higher education, students, recommendation compliance, mental health, academic self-efficacy, digital interventions

## Abstract

**Aims:**

The COVID-19 containment strategy in Sweden uses public health recommendations relying on personal responsibility for compliance. Universities were one of few public institutions subject to strict closure, meaning that students had to adapt overnight to online teaching. This study investigates the prevalence of self-reported recommendation compliance and associations with self-reported symptoms of contagion, self-experienced effects on mental health and academic self-efficacy among university students in Sweden in May–June 2020.

**Methods:**

This was a cross-sectional 23 question online survey in which data were analysed by multinomial regression, taking a Bayesian analysis approach complemented by null hypothesis testing.

**Results:**

A total of 4495 students consented to respond. Recommendation compliance ranged between 70% and 96%. Women and older students reported higher compliance than did men and younger students. Mild to moderate COVID-19 symptoms were reported by 30%, severe symptoms by fewer than 2%; 15% reported being uncertain and half of the participants reported no symptoms. Mental health effects were reported by over 80%, and changes in academic self-efficacy were reported by over 85%; in both these areas negative effects predominated. Self-reported symptoms and uncertainty about contagion were associated with non-compliance, negative mental health effects, and impaired academic self-efficacy.

**Conclusions:**

Students generally followed public health recommendations during strict closure of universities, but many reported considerable negative consequences related to mental health and academic self-efficacy. Digital interventions should be developed and evaluated to boost coping skills, build resilience and alleviate student suffering during the pandemic and future similar crises.

## Introduction

The COVID-19 pandemic has led to unprecedented restriction of individual movement, daily routines and economic activity. Such restrictions have varied between countries in the degree of coercion directed towards ordinary citizens, and it is unclear whether differences in the severity of measures have affected the spread of the pandemic; these circumstances have challenged thinking about public health actions on the individual, societal and governmental levels [1]. A recent rapid review of existing research on psychological aspects of pandemic-related quarantine indicated that negative long-term effects most often ensue, but that presenting a clear official rationale for restrictions that are voluntary, rather than mandatory, can mitigate the psychological distress associated with quarantine [2, 3]. Awareness among public health authorities of known mental health challenges in relation to the pandemic has been signalled through recommendations on mental health, issued by the World Health Organization (WHO) [4] and by the Public Health Agency of Sweden (PHA) [5]. A call for action on COVID-19-related mental health issues has further recommended mapping mental health effects, evaluating coping and resilience mechanisms, and designing interventions that can quickly be put in place to promote maintenance of mental health and prevent its decline [6].

Sweden is a country which rates low in relation to most other European countries on the ‘freedom restriction index’ [7]. The Swedish PHA has recommended restrictions to limit contagion in the entire population, focusing primarily on physical distancing, avoiding public transportation as well as all unnecessary international travel, regular hand-washing, sneezing or coughing in one’s sleeve, avoiding socialisation with individuals in specified risk groups, including those 70 years of age and over, working at home when possible and remaining at home at the least sign of symptoms of contagion [8]. No mandatory enforcement regulations or sanctions have been attached to the recommended restrictions. Compliance with voluntary restrictions can, however, be expected to be high in Sweden, a liberal society where trust in government is relatively high [9], and emphasis on individual responsibility for personal health is also high [10]. A study on pandemic effects on mental health in the adult population in Sweden nonetheless indicated prevalences of 30% for symptoms of depression, 24.2% for anxiety and 38% for insomnia, clearly higher rates compared to national survey results from the Swedish general population before the pandemic that showed rates of 10.8% for depression, 14.7% for anxiety and 7–10% for insomnia [11]. Earlier research from the severe acute respiratory syndrome (SARS) pandemic in Canada indicated that social restrictions in the form of home quarantine were associated with post-traumatic stress disorder (PTSD) and depression symptoms of clinical significance among about one-third of the general population [12], in alignment with current findings in Sweden [11]. Of relevance is also that other broad societal changes that occur during a pandemic can negatively affect mental health; for example, economic recession is particularly associated with upward trends in the prevalence of suicides [13].

Students at the university and college levels generally comprise a large group in national populations, specifically almost 350,000 individuals in Sweden, equivalent to about 3.5% of the total population or 26% of the population between 20 and 30 years old in 2020 [14]. As the COVID-19 pandemic emerged, significant changes occurred in students’ study situation in Sweden. On 17 March 2020, the PHA declared that no public meeting with over 50 participants could be held. This led to a nationwide decision to eliminate physically present teaching on university premises, and Swedish universities and colleges transitioned to online teaching literally over one night on 18 March 2020, a situation that has persevered during the autumn and spring terms of the 2020–2021 academic year. The decision to prohibit university students en masse from attending classes may have led to greater challenges in complying with pandemic-related restrictions, and also to more severe consequences on student mental health than for adults in the general population in Sweden.

One reason for possibly greater pandemic-related negative mental health effects among university students is also that they may find themselves in a particularly vulnerable situation in view of their status as emerging adults engaged in a process of forming their own professional identities [15]. Internationally, about one-fifth of university students have been shown to suffer from current mental health issues [16], and over one-third of first-year students have been identified with at least one mental disorder [17]; in both cases, these data are based on diagnostic algorithms calculated from online self-reported responses to diagnostic criteria. In Sweden, public health research based on self-reported signs and symptoms, measures that are more inclusive than diagnostic criteria, shows that between 25% and 50% of students show signs of mental ill-health [18]. Although the Swedish prevalence of mental health problems may seem high, it is well in accordance with the cited international data based on more stringent calculation of diagnostic algorithms.

General threats posed by the pandemic include physical effects of infection, increased sedentary behaviour coupled with a reduction in physical activity [19], mental health effects including loneliness [20], as well as effects on everyday working life [21] and studies. Investigating the current impact of the COVID-19 pandemic, in terms of recommendation compliance and its associations with self-reported physical symptoms, mental health and academic self-efficacy among university students in Sweden is of high priority, in order to understand ongoing trends as well as to provide information that can be used to design and deliver interventions to support students as they continue personally to manage the restrictions imposed by the pandemic. Given that these young adults form the bulwark of the social fabric in the coming decades, this work is vital to understanding effects in this group on the current and future sense of continuity regarding their lives. Such a sense of continuity is part of the key concept of ontological security, which also relates to a sense that one possesses ‘answers to fundamental existential questions which all human life in some way addresses’ [22, p. 47].

Published research on university students’ recommendation compliance in relation to physical and mental health during the COVID-19 pandemic is continually increasing [23]. Early research relevant to this study reported that younger persons and men were less likely to comply with recommendations; in terms of mental health, women, younger persons under 30 years of age and those having student status had a higher risk of anxiety and depression [24]. A recent publication on predictors of student compliance with restrictions in Denmark showed an overall level of 68% compliance, in which high compliance was predicted by factors such as being an older student or a PhD candidate, as well as being concerned about contagion and feeling depressed. Lower compliance was predicted by living in a dormitory, feeling stressed and drinking over seven standard drinks a week [25]. Early research among students regarding mental health during the pandemic has shown that social support protected against anxiety among 7143 university students in China [26]. Also, higher levels of ‘fear of COVID-19’ were found among students who were part of a cohort of 850 young adults in Russia and Belarus [27]. Symptoms of mild to severe depression were found among over 80% of 2031 university students in the USA, with over 70% showing anxiety at similar levels [28]. These examples suggest that student mental health is significantly affected by the pandemic, and raise the question of how mental health effects might differ by country. In Sweden, no research specifically targeting students during the pandemic has been published at this writing, to the best of our knowledge. Some indication of student mental health in Sweden has come from the above-cited study on mental health impacts, which showed that student status (29.1% of the sample) had positive significant associations with scores on the depression, anxiety and insomnia measures used [11]. The COVID-19 International Student Well-being Study has also collected data from students in Sweden but has not yet published the results [29].

## Aims

The current study was conceived in answer to a call to a general action for research on student mental health in the pandemic [30] and in connection with our surveys of student mental health data within the WHO World Mental Health–International College Student Initiative (WMH-ICS) [31]. Our purpose here is to collect data on the extent to which public health recommendations to limit the spread and contagion of the COVID-19 virus have been followed by students at Swedish universities and colleges, and to investigate associations between these reports and self-experienced effects on students’ physical health, mental health and academic self-efficacy. Investigating these variables is important in view of the varied responses that pandemic-related restrictions may elicit in terms of compliance, and in view of research suggesting that the existence of these restrictions is related to heightened levels of mental health symptoms, perhaps particularly so among students in higher education. Our primary research questions are: (a) What is the overall prevalence of recommendation compliance, including differences by demographic factors?; (b) What are the prevalences of self-reported symptoms of COVID-19 contagion, self-reported effects on mental health and academic self-efficacy; (c) To what extent is recommendation compliance associated with self-reported symptoms; and (d) To what extent are self-reported symptoms associated with self-reported mental health effects, and self-reported academic self-efficacy effects?

## Methods

In this cross-sectional study, an online survey was disseminated to students at 10 specified universities and colleges in Sweden; it was also accessible to students at additional institutions of higher education via the National Student Union Association website. The survey was designed for this research and was available in Swedish and English. The full survey included 23 questions with 10 follow-up questions dependent on display/skip logic (e.g. those reporting only negative health effects were asked no follow-up questions about positive effects). Individual respondents thus answered between 13 and 23 questions. The questions covered five areas: (a) demographic questions; (b) experiences related to COVID-19 (extent of following recommendations; if not followed, why not); (c) personally experienced virus symptoms; (d) mental health effects of the pandemic; and (e) effects on respondents’ academic self-efficacy, and satisfaction with university’s pandemic management. The authors designed the study questionnaire, with student representation from co-author LG to increase survey face validity. A summary of all questions with response alternatives is available in our pre-registered analysis plan [32].

### Ethics

Ethical approval was granted (ref. no. 2020-02109) by the Swedish Ethical Review Authority. Respondents were free to contact the researchers to ask any questions, at a generic survey address monitored on weekdays by the first author, or via e-mail addresses and mobile phone numbers to the first and last authors. If needed, respondents were referred to appropriate treatment services according to a prepared list of nationally available services or to local student mental health services.

### Procedure

The survey targeted students at 10 participating state universities. Students were recruited via local communication plans at their respective university; that is, via advertisements on the university website, social media and, at one university, via direct e-mails including an anonymous link to the survey. This meant a potential sample size of 169,412 registered students, or 49.4% of the first and second-cycle 343,080 students registered in the spring term of 2020 at any of 45 accredited institutions of higher education in Sweden [33]. Additional recruitment took place via advertisement on the website of Sweden’s United Student Union, and was accessible to any student. The questionnaire remained open between 18 May 2020 and 25 June; the start date was exactly 2 months to the day after in-person teaching in institutions of higher education was cancelled in Sweden in favour of online teaching. Students accessed the survey via a direct, anonymous URL or QR-code, where detailed project information was available. A question concerning informed consent was provided online directly after the project information. Providing consent led immediately to the survey questions, while lack of consent led to survey termination.

### Sample

A total of 4495 individuals consented to participate, the great majority from the 10 participating universities, with participants from other universities numbering under 50. Of the total sample, 70.9% were women, with a mean age of 26.5 years (standard deviation 5.27), coming from 19 Swedish universities/colleges, distributed across the following areas of study: social sciences 19.2%, humanities 14.2%, medicine and dentistry 10.5%, health sciences 9%, natural sciences 8.6%, technical sciences 7.8%, fine arts 6.7% and other 24.2%. The ‘other’ category included educational programmes that were combinations of the overriding study categories used, or students enrolled in more than one parallel educational programme. Our intention was to include a text field to clarify ‘other’ areas of study, but due to a technical error the text field was omitted.

### Statistical analysis plan

A full statistical analysis plan was registered after the data were collected, but prior to downloading the data from the online survey system. We present only the core of the analysis plan; the full plan is available elsewhere (https://osf.io/gd2z6/). Each research question was addressed by estimating associations among variables using multinomial regression. For instance, for the second research question examining associations between compliance with recommendations and self-reported COVID-19 symptoms, a multinomial regression model was estimated with self-reported symptoms as the dependent variable and the seven compliance recommendation items as independent variables. Models also included age and gender as covariates.

All models were estimated using Bayesian inference with standard normal priors and regularising priors [34, 35]. The Bayesian approach allows for calculating a distribution over model parameters which shows their compatibility with the data, and thus allows for calculating the probability of different parameter values. For instance, it allows for calculating the probability that an odds ratio is greater or less than 1 given the collected data. In contrast, the frequentist approach usually outputs a single maximum likelihood estimate (MLE) which tells which model parameter values make the data most likely. MLE estimates are sensitive to single data points, and null hypothesis tests based on these are likewise very sensitive, making them unreliable [36]. An additional benefit of using Bayesian inference is the ability to incorporate prior information into the analyses. This stabilises estimates and makes them less sensitive to single data points, and also allows for regularisation of the model parameters.

We used standard normal priors for the primary analyses, which a priori indicate that we believe most log odds ratios to be centred around 0. To aid in the identification of covariates associated with the dependent variables, we also used regularising priors [35], a strategy which encodes a very strong a priori belief that covariates are 0, effectively pulling all covariates to 0 unless the data strongly suggest otherwise. This approach allows for calculating a distribution over model parameters, and protects from issues arising from having several covariates in the model. We complemented our Bayesian analyses with MLE estimates and null hypothesis tests.

## Results

**Figure 1.**
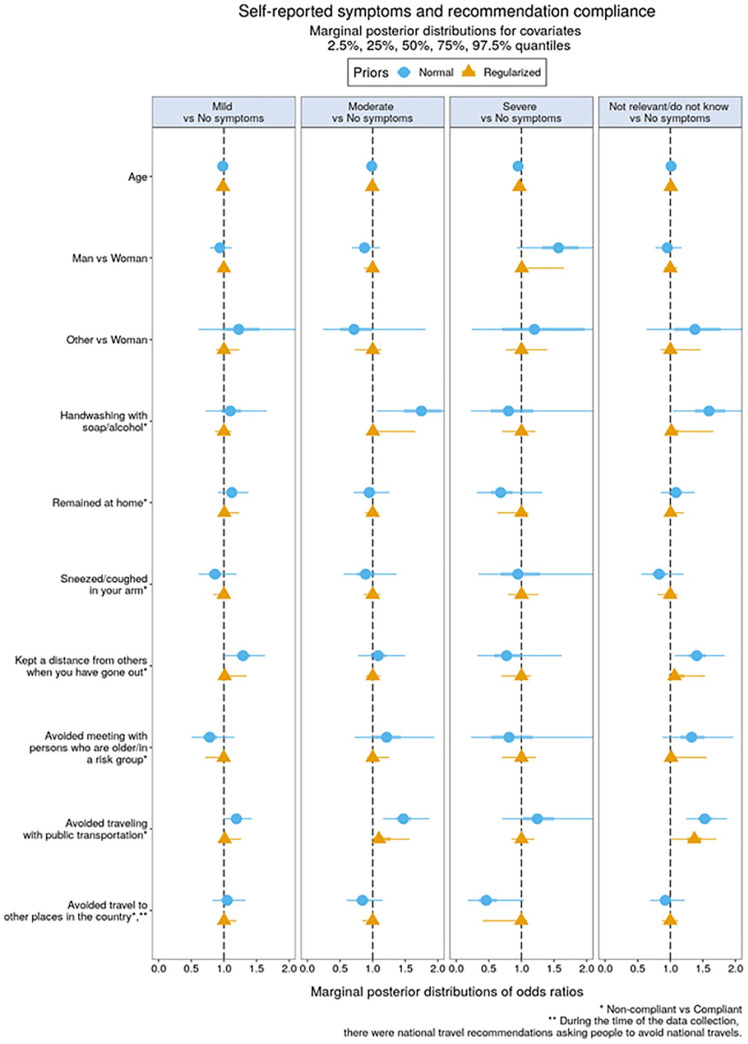
Recommendation compliance versus self-reported somatic symptoms.

**Figure 2.**
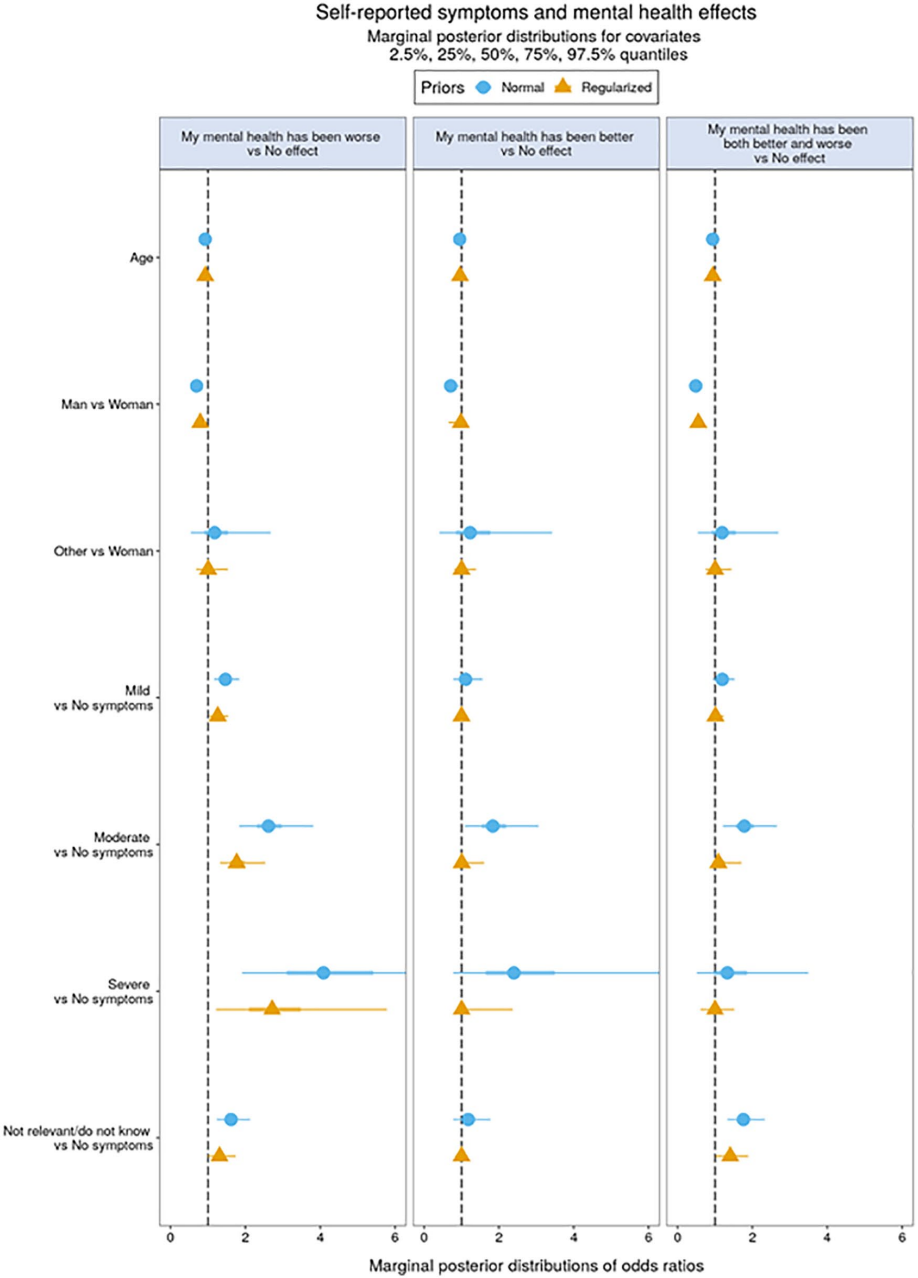
Self-reported symptoms versus mental health effects.

**Figure 3.**
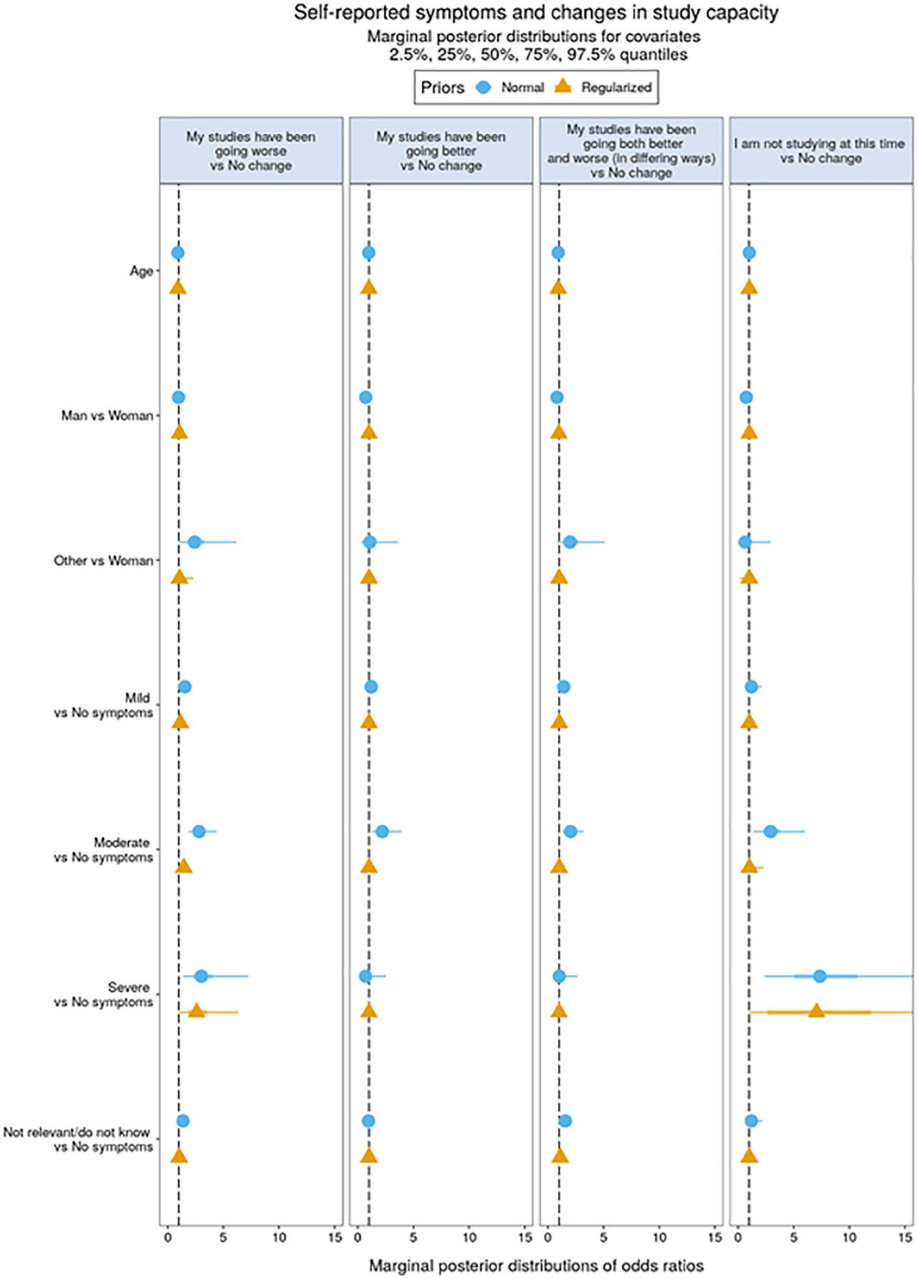
Self-reported symptoms versus changes in academic self-efficacy.

Results are reported in order of the research questions, with reference to figures and tables within the manuscript (Figures 1–3; Tables I–III) and to Supplemental Material summarising the data output (Supplemental Figures 1–3 and Supplemental Tables 1–12).

**Table I.** Specific negative phenomena reported by 2812 survey respondents experiencing worse or both worse and better mental health, 92% entirely or partly attributed to pandemic (multiple responses possible).

Phenomena	*N*	Percentages
Total number of responses on symptoms,^a^ ranked by %	6119	% of *N*
Worry or anxiety	1817	29.7%
Stress	1603	26.2%
Depression or low mood	1559	25.5%
Difficulty sleeping	824	13.5%
Other^c^	316	5.2%
Total number of responses on worse aspects,^b^ ranked by %	10,077	% of *N*
Difficulty studying	1929	19.1%
Lonely	1696	16.8%
Worry about others	1558	15.5%
Worry about society	1543	15.3%
Worry about finances	1185	11.8%
Worry about studies	1047	10.4%
Worry about getting ill	721	7.2%
Other^c^	255	2.5%
Being in a risk group	143	1.4%

a Q10: You indicated that your mental health during the past 4 weeks has been worse, and that this worsening is entirely or partly due to the COVID-19 pandemic. Which mental health symptoms have you had as a result of the COVID-19 pandemic? (You may indicate several responses.)

b Q11: You indicated that your mental health during the past 4 weeks has been worse, and that this worsening is entirely or partly due to the COVID-19 pandemic. In what way/s has the COVID-19 pandemic worsened your mental health? (You may indicate several responses.)

c Text response option available. Text analysis will be presented elsewhere.

**Table II.** Specific positive phenomena experienced by 1408 survey respondents experiencing better or better and worse mental health, 59% entirely or partly attributed to pandemic (multiple responses possible).

Phenomena	*N*	Percentages
Total number responses on symptoms,^a^ ranked by %	1258	% of *N*
I feel calmer, less stressed	419	33.3%
Other^c^	309	24.6%
Happier or more satisfied than usual	209	16.6%
Improved sleep	199	15.8%
Not been as worried as usual	122	9.7%
Total number responses on better aspects,^b^ ranked by %	2052	% of *N*
Felt privileged as a student	550	26.8%
Stayed at home and felt less stressed	509	24.8%
Easier to study at home/remotely	291	14.2%
My problems seem less severe in light of the crisis	188	9.2%
Meaningfulness in doing good works (relative to pandemic)	187	9.1%
More feeling of community and togetherness	180	8.8%
Other^c^	147	7.2%

a Q13: You indicated that your mental health during the past 4 weeks has been better, and that this improvement is entirely or partly due to the COVID-19 pandemic. Which mental health symptoms have you had as a result of the COVID-19 pandemic? (You may indicate several responses.)

b Q14: You indicated that your mental health during the past 4 weeks has been better, and that this improvement is entirely or partly due to the COVID-19 pandemic. In what way/s has the COVID-19 pandemic improved your mental health? (You may indicate several responses.)

c Text response option available. Text analysis will be presented elsewhere.

**Table III.** Specific effects on academic self-efficacy experienced by 2918 survey respondents experiencing worse academic self-efficacy or both worse and better self-efficacy (multiple responses possible).

Effects	*N*	Percentages
Total number specific responses on worse effects,^a^ ranked by %	9463	% of *N*
Harder for me to concentrate on my studies	2064	21.8%
Less contact with fellow students affects my studies negatively	1958	20.7%
Studying at home is hard for me	1551	16.4%
Online teaching has not worked well for me	982	9.7%
Worry about not completing education in time affects studies negatively	649	6.9%
My university/college has not adapted teaching so that it works well	554	5.9%
Worry about my own or other’s health affects studies negatively	496	5.2%
Other^b^	289	3.1%

a Q16: You indicated that your studies have been negatively affected by the COVID-19 pandemic. In what way/s has the pandemic affected your studies negatively? (You can indicate several answers.)

b Text response option available. Text analysis will be presented elsewhere.

### Overall prevalence of recommendation compliance including differences by demographic factors

Recommendation compliance was overall very high for hand-washing (95.7%), avoiding risk groups (95.5%), and sneezing/coughing in one’s sleeve (93.7%), high for maintaining physical distance (87.2%), avoiding travel across Swedish regions (both public and private; 86.6%), and staying at home (81.2%), but moderate for avoiding public transportation (69.7%), see Supplemental Table 1.

Regarding demographic factors, shown in Supplemental Table 8, women students were more likely to comply with recommendations than men, specifically for sneezing or coughing in their sleeve, maintaining physical distancing when outside the home, avoiding encounters with individuals in risk groups, and avoiding national travel across Swedish regions. Younger students 16–25 years old were less likely than students in the middle range of 25–35 years to comply with recommendations to remain at home, maintain physical distancing and avoiding national travel. However, younger students were more likely than older students to comply with sneezing or coughing in their sleeves. Older students aged 36 years and over, compared to 16–25-year-olds, showed higher rates of compliance in relation to maintaining physical distancing, avoiding public transportation, and avoiding travel to other parts of the country, but were less compliant regarding remaining at home, sneezing/coughing in their sleeves and avoiding meeting with older persons or those in a risk group (see Supplemental Table 10). In general, differences in recommendation compliance between age groups were related to limiting social contacts.

Regarding differences between universities, the largest group in the sample came from a university in the second largest city in Sweden. Students at this university reported higher compliance regarding physical distancing, avoiding encounters with older people or those at risk and avoiding national travel across Swedish regions, compared to those at the other participating universities (apart from the Royal College of Music, which was analysed separately, see Supplemental Tables 11 and 12). At the same time, these same students reported lower compliance with the recommendation to avoid public transportation. These contrasts may relate to the metropolitan location of the students in this large group.

Supplemental Figures 1–3 show the marginal posterior distributions of coefficients in the multinomial regression model representing associations between recommendation compliance and gender, age and university, respectively. Overall numerical details are given in Supplemental Tables 7–12.

### Prevalence of self-reported somatic symptoms and self-reported mental health and academic self-efficacy effects

Self-reported somatic symptoms of COVID-19 were experienced by 35% of the respondents, 14.9% reported uncertainty regarding whether that had experienced COVID-19 symptoms and 50.1% reported no symptoms. Of the 35% who experienced symptoms, 65.8% reported mild somatic symptoms, 29.6% reported experiencing moderate symptoms and 4.6% reported severe symptoms, corresponding to 23.1%, 10.4% and 1.6% of the entire sample, respectively, see Supplemental Table 1.

Self-experienced changes in mental health due to the pandemic were reported by over 80% of respondents, with negative effects experienced by 43.7% and both better and worse effects experienced by 30.4%. Regarding worse effects reported by both these groups (*n*=2812; see Table I), general worry or anxiety, stress and depression or low mood were reported in over 25% of the total responses, when multiple responses were possible (*n*=6119). Top-ranked specific worse aspects were difficulty studying (19.1%) and feeling lonely (16.8%), followed by worries about others, society, finances, studies and falling ill, in descending order; multiple responses were possible here too (*n*=10,077).

Positive effects that were attributed to the pandemic were experienced by 7.7% of respondents and were also reported by the 30.4% of respondents experiencing both better and worse effects (*n*=1408; see Table II). Positive effects included feeling calmer and less stressed (33.3%), ‘other’ positive effects (24.6%), feeling happier or more satisfied than usual (16.6%) and better sleep (15.8%); multiple responses were possible (*n*=1258). Top-ranked specific better aspects included feeling privileged as a student (26.8%), staying at home and feeling less stressed (24.8%), followed by finding it easier to study at home, viewing one’s own problems as less severe in light of the pandemic, and finding it meaningful to be helpful and feeling a sense of community and togetherness, in descending order (multiple responses possible; *n*=2052).

Changes in academic self-efficacy were experienced by over 85% of the respondents, with 43.6% experiencing worse academic self-efficacy, 34.6% experiencing both better and worse effects and better effects by 6.8%. A small proportion of respondents (2.4%) also reported that they were not studying at this time and did not comment on any academic self-efficacy effects. Among the 78.2% (*n*=2918) who experienced some kind of worse effects (multiple responses possible; *n*=9463; see Table III), specific effects included finding it harder to concentrate on studies (21.8%), finding that less contact with fellow students affected studies negatively (20.7%) and experiencing home studying as difficult (16.4%). Also 9.7% of the responses indicated that online teaching had not worked for the respondent, followed by worry about not completing education in time, experiencing that the educational institution had not adapted teaching so that it works well, worry about one’s own or others’ health and ‘other’, in descending order.

### Associations between recommendation compliance and self-reported somatic symptoms

Figure 1 shows the marginal posterior distributions of coefficients in the multinomial regression model representing associations between recommendation compliance and self-reported somatic symptoms. Lines with circle icons represent analyses using standard normal priors, and lines with triangle icons represent analyses using regularising priors (see Supplemental Tables 1 and 2 for numerical details). Non-compliance with avoiding public transportation was markedly associated with moderate somatic symptoms, as it was not shrunk in the regularising model, as were the responses of ‘not relevant/do not know’. This suggests that clear experience of moderate somatic symptoms, as well as uncertainty about having been infected, both seemed related to facing unknown others in the more crowded public situations typical of travel with public transportation.

### Associations between self-reported somatic symptoms and effects on mental health and academic self-efficacy

Respondents reporting mild, moderate and severe symptoms, as well as those choosing the ‘not relevant/do not know’ response, were more likely to report negative mental health effects than those with no symptoms. Responding that mental health was both better and worse was markedly associated with moderate symptoms and responding ‘not relevant/do not know’. Figure 2 shows the marginal posterior distributions of coefficients in the multinomial regression model representing associations between self-reported symptoms and effects on mental health. Full numerical details are shown in Supplemental Tables 3 and 4. The Bayesian marginal posterior distribution suggests that worse mental health was associated with any level of infection with the COVID-19 virus as well as with being uncertain about one’s own contagion (see orange-shaded rows in Supplemental Table 4).

A similar pattern emerged in relation to negative effects on academic self-efficacy, which was associated with somatic symptoms ranging from mild to severe. In addition, reporting both positive and negative effects on academic self-efficacy was associated with responding ‘not relevant/do not know’ concerning somatic COVID-19 symptoms. Overall, lower academic self-efficacy was related to having been infected in some way, or being uncertain about this. Not currently being a student was associated with having severe somatic symptoms, suggesting severe symptoms obliged respondents to cease studying. Figure 3 shows the marginal posterior distributions of coefficients in the multinomial regression model representing associations between self-reported somatic symptoms and effects on academic self-efficacy; see Supplemental Tables 5 and 6 for full numerical details.

## Discussion

In summary, our findings showed that students in Sweden seem generally to have complied with recommendations to restrict their movements, and they have also changed their daily hygiene-related behaviours. Men and younger students had more difficulty with compliance than did women and older students, in line with recent findings [24]. Half of the respondents had not experienced any somatic symptoms, and about a third had experienced mild to moderate symptoms, with a small proportion experiencing severe symptoms and the remaining respondents being uncertain about contagion. Higher rates of contagion were associated with lower self-reported compliance with recommendations to avoid public transportation. The prevalences of self-reported negative effects on mental health and academic self-efficacy were high, and experiencing negative effects in both these areas was markedly associated with mild to moderate self-reported symptoms or uncertainty about contagion. Severe symptoms were associated with no longer being a student; it should be noted that no causal relationship between symptom severity and study termination can be concluded.

Compliance rates among students in Sweden for all measures recommended by the government were higher than the overall rate of 68% reported among students in Denmark, where compliance was measured by a single question asking to what extent respondents adhered to government restrictions [25]. This finding aligns with differences between Sweden and Denmark regarding the ‘freedom restriction index’, where Sweden rates twice as low as Denmark [7], meaning that individual citizens’ freedom is less restricted in Sweden compared to Denmark and that the explicitly voluntary nature of recommendations in Sweden may have facilitated increased compliance [2, 3]. Only when it came to avoidance of public transportation were compliance rates somewhat lower in Sweden at just under 70%, although still higher than overall self-reported adherence among students in Denmark. The lower compliance in Sweden with avoidance of public transportation was markedly associated with moderate symptoms of self-reported somatic symptoms, suggesting analogously that avoidance of public transportation could be a significant protective factor against contagion.

We address some possible concerns about the representativeness of our sample before turning to a discussion on the implications of our findings. Our sample included 70% women; however, the majority of individuals in the population 25–34 years old with university educations of 3 or more years are women (62%) [37] – close to the proportion in our sample. Although women have been found to be more likely to follow recommendations for restrictions during the COVID-19 pandemic, associated with a perception of higher threat as well as lower trust in health services [38], we find that the risk of biased findings in relation to the overrepresentation of women in the sample is low. Moreover, our findings follow earlier findings showing that men had more difficulty complying with recommendations than women [24]. Another question concerns the degree of overall representativeness in our sample. We approached students via 10 separate universities as well as via Sweden’s United Student Unions, resulting in reaching students at 16 of the 45 accredited institutions of higher education in Sweden. University communication strategies for presenting the study to their students varied widely. Most universities posted advertisements on their websites, but did not amass a concerted effort to reach potential respondents. However, one of the universities applied a well-developed communication strategy, which was very successful in that 71% of our respondents came from there. The response rate in relation to all students at that particular university (*n*=34,770) was 8.7%. A possible disadvantage here is that the overrepresentation of students from one university, in a large urban context, may have biased the sample. The potential total sample size of 169,412 registered students at the 10 formally participating universities where students were recruited constitutes about 49.4% of the first and second-cycle 343,080 registered students in the spring term of 2020 at 45 accredited higher education institutions in Sweden [33]. Our sample size of 4495 students was thus equivalent to approximately 2.7% of the potential sample. Still, we recruited a large sample, larger than in the most closely comparable published study concerning student recommendation compliance in Denmark, where 2945 students were recruited, with response rates of 10–18% in subsamples directly targeted via e-mail, and an unknown response rate among an additional directly approached subsample as well as Facebook group recruits [25]. Our findings also align with already published research on student pandemic-related experiences during the spring of 2020 [11, 25–28, 39]. We suggest that it is thus meaningful to discuss the implications of our findings for future pandemic management by university leadership as well as for students themselves.

Overall, the survey results indicate that although students are generally compliant with pandemic-related recommendations, they are struggling to maintain their mental health and academic self-efficacy amid the pandemic restrictions. These findings have implications on policy, service and research levels. From policy and service perspectives, university leadership, mental health services and student unions need to address the struggles experienced by students in a constructive way. Universities have managed the practical challenges of the pandemic by arranging for studies to continue, albeit remotely, and supported students in following recommendations by informing them continually about the need to follow recommendations and maintain caution. From a service point of view, existing student health services are the only resources available in terms of helping students manage their emotional state, and anecdotal evidence from Sweden indicates that the capacity of existing student health services has been significantly stretched (I Dahlgren, personal communication, spring 2021).

From a research perspective, knowledge gaps exist regarding how best to support students during global crises such as the COVID-19 pandemic. To our knowledge no novel supportive interventions have been developed and delivered to university students within this short period of time, although a recent rapid meta-review suggests that digital interventions could be well aligned with the need to address the psychological impact of the pandemic on a population-based level including students, particularly from a mental health promotion perspective [40]. One concept easily recognised by students is stress, experienced in relation to their studies, examination, transitioning to the university environment and financial issues [41]. The links are also clear between stress and the incidence of most diseases, particularly depression [42]. A source of inspiration for intervention development can therefore involve stress prevention programmes, which have led to mild to moderate reductions in stress levels, depression and anxiety symptoms among students in higher education [43]. The mechanism by which such programmes contribute to successful prevention is related to stress resilience, key to overall wellbeing, which can protect participants from developing mental health problems such as depression [44]. Building this kind of resilience could help mitigate the effects of ongoing societal problems such as the still ongoing pandemic, as well as future challenges. Delivering such programmes digitally could be an effective way of supporting a large group of students, as digital interventions for mental health issues have been shown to be equally effective as face-to-face intervention [45].

Our findings show that being directly affected by mild to moderate somatic symptoms, or being uncertain about whether infection has occurred, carried a higher risk of negative effects on mental health and academic self-efficacy. This group would benefit from targeted interventions to mitigate worries derived from having been ill, based on stress prevention programmes that would include a peer-support component for supporting the practice of newly acquired – or boosted – skills [46]. Students who have not experienced any somatic symptoms and are concerned about becoming infected might benefit from interventions targeting such concerns. One such intervention, a 3-week self-help digital intervention based on cognitive-behavioural therapy (CBT) and developed to mitigate pandemic-related specific worries in adults, has shown positive post-intervention effects on anxiety reduction and is freely available via the healthcare system in three large urban regions in Sweden [47].

Our findings also have conceptual implications for understanding the psychological impact of the pandemic, with regard to the type of negative emotions experienced by university students in this crisis. We found that a large proportion of students who specified what sort of worse mental health effects they experienced chose worry, stress and low mood to describe their feelings, and also said they had trouble studying, felt lonely and worried about a variety of pandemic-related issues. Although positive effects were reported, these were considerably fewer than the negative effects. Academic self-efficacy was also negatively affected among three-quarters of the respondents. This suggests that the students may have construed the pandemic and the sudden lockdown of on-campus university studies as introducing high uncertainty regarding their education, and found this quite challenging to tolerate. Explicit uncertainty in situations of both high and low threat has been shown to provoke more anxiety than implicit uncertainty, in an experimental study among undergraduates [48]. The situation generated by the pandemic can be described as generating explicit uncertainty on a day-to-day basis over at least the 3-month period from March to June 2020, relevant for this study. The level of threat has been unclear, and might be lower for younger people as they have been defined as less susceptible to contagion than older people 70 years and above in the population. Nonetheless, the explicit uncertainty about this multitude of factors may have been a causal factor for the worse mental health and academic self-efficacy reported by our respondents. An additional research question is to what extent pandemic-related somatic and emotional experiences might connect with life narratives of health and illness, issues that could be addressed in cross-cultural research from anthropological, sociological and psychological perspectives in order to explore how to promote the building of resilience among university students in the face of future similar events [49, 50].

This study had several strengths as well as some limitations. One strength was that the survey was launched just 2 months after strict closure of universities began. Secondly, information about the survey was disseminated among 10 Swedish universities accounting for about half of all registered students and reached a sample of over 4000 participants. Thirdly, the focus of the study was compliance, self-reported somatic symptoms, effects on mental health and on academic self-efficacy, yielding an overview of associations between these factors. Study limitations include a risk of selection bia, with a large number of respondents from one of the included universities, a lack of clarity regarding the overall representativeness of the sample (see above), self-report methods, the original design of the survey and the cross-sectional design. Although self-reports carry a risk of common method variance, with possible overestimation of associations [51], our analytical approach is conservative, using weakly and strongly informative priors of no association, mitigating to some degree the effects of possible bias away from the null; also, individuals are best placed to describe their own behaviours and experiences [51]. Regarding the survey design, this seems to have had high face validity for respondents, but carried the limitation of not using already validated existing scales allowing direct comparison to previous research, at least for mental health issues, as was done in an adult Swedish sample [11]. Given that our primary focus was on associations between compliance rates and self-reported symptoms, mental health and academic self-efficacy effects, and that the prevalence of mental health issues was high and aligned with other similar research, this limitation may not have had any significant negative consequences for the validity of our findings and conclusions, allowing for valuable insights regarding compliance, mental health and academic self-efficacy among students in Sweden.

## Conclusions

In conclusion, this study contributes to the initial evaluation of whether closing universities and colleges may be a constructive strategy for managing possible future pandemics, as well as providing information on what types of psychological and educational needs may exist among students in the continuation of the pandemic and other similar crises. Clearly, supportive interventions in digital format for the promotion of mental health and prevention of stress among university students need to be further developed and evaluated to increase both short and long-term effectiveness, as has been suggested in recent meta-analyses [52, 53]. Further avenues for research should include analysis of findings from longitudinal data collection among university students who have experienced higher education during the COVID-19 pandemic, with a particular focus on possible changes in resilience to stress. Finally, a vital additional area requiring more research is the investigation of whether having a history of mental health issues is related to increased negative psychological impact of the pandemic, and how to respond adequately to the needs of this group [39]. All these research questions require the research community’s urgent attention.

## Supplemental Material

sj-docx-1-sjp-10.1177_14034948211027824 – Supplemental material for Compliance with recommendations limiting COVID-19 contagion among university students in Sweden: associations with self-reported symptoms, mental health and academic self-efficacy Click here for additional data file. Supplemental material, sj-docx-1-sjp-10.1177_14034948211027824 for Compliance with recommendations limiting COVID-19 contagion among university students in Sweden: associations with self-reported symptoms, mental health and academic self-efficacy by Anne H. Berman, Marcus Bendtsen, Olof Molander, Petra Lindfors, Philip Lindner, Lilian Granlund, Naira Topooco, Karin Engström and Claes Andersson in Scandinavian Journal of Public Health

sj-docx-2-sjp-10.1177_14034948211027824 – Supplemental material for Compliance with recommendations limiting COVID-19 contagion among university students in Sweden: associations with self-reported symptoms, mental health and academic self-efficacy Click here for additional data file. Supplemental material, sj-docx-2-sjp-10.1177_14034948211027824 for Compliance with recommendations limiting COVID-19 contagion among university students in Sweden: associations with self-reported symptoms, mental health and academic self-efficacy by Anne H. Berman, Marcus Bendtsen, Olof Molander, Petra Lindfors, Philip Lindner, Lilian Granlund, Naira Topooco, Karin Engström and Claes Andersson in Scandinavian Journal of Public Health
