# Supplementary material for: Compliance with recommendations limiting COVID-19 contagion among university students in Sweden: associations with self-reported symptoms, mental health and academic self-efficacy
Source: Scand J Public Health. 2021 Jul 2;50(1):70–84. doi: 10.1177/14034948211027824 (PMC8808007; doi:10.1177/14034948211027824)
Supplement: sj-docx-1-sjp-10.1177_14034948211027824 – Supplemental material for Compliance with recommendations limiting COVID-19 contagion among university students in Sweden: associations with self-reported symptoms, mental health and academic self-efficacy [file sj-docx-1-sjp-10.1177_14034948211027824.docx]

# Supplementary figures


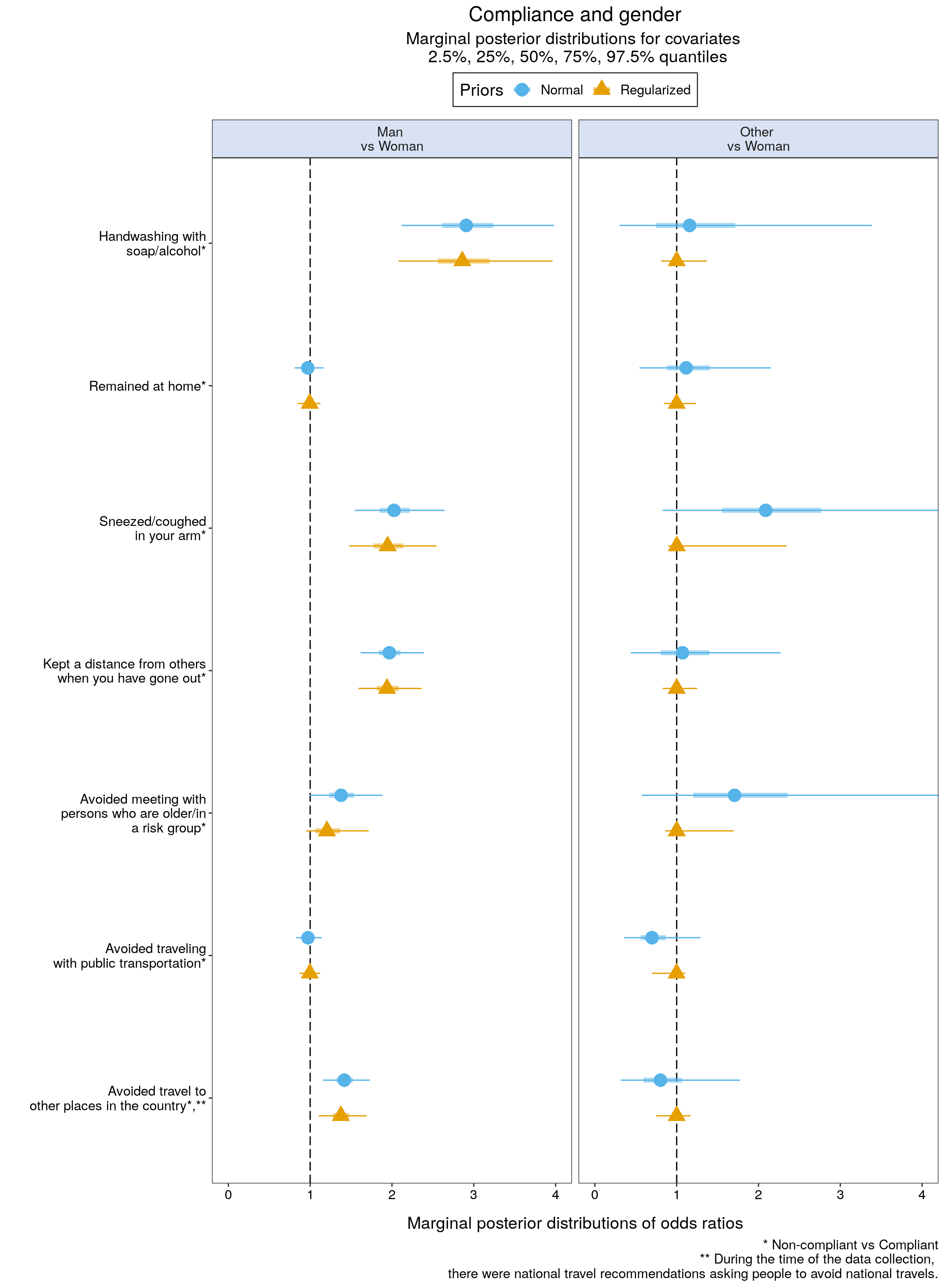


Figure S1 - Marginal posterior distributions using standard normal priors and regularizing priors (censored at OR = 4) – Recommendation compliance and gender.


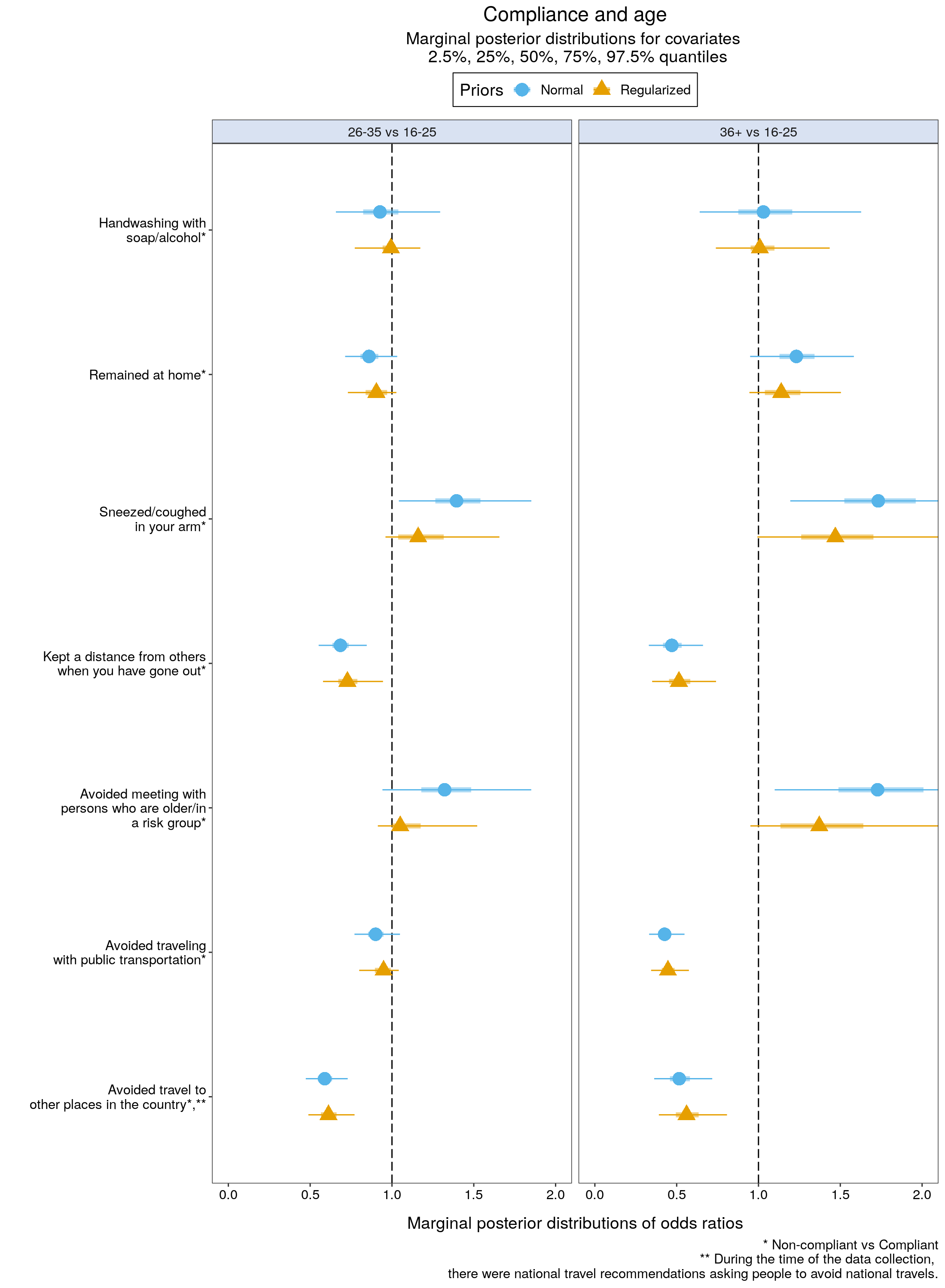


Figure S2 – Marginal posterior distributions using standard normal priors and regularizing priors (censored at OR = 2) – Recommendation compliance and age (pooled).


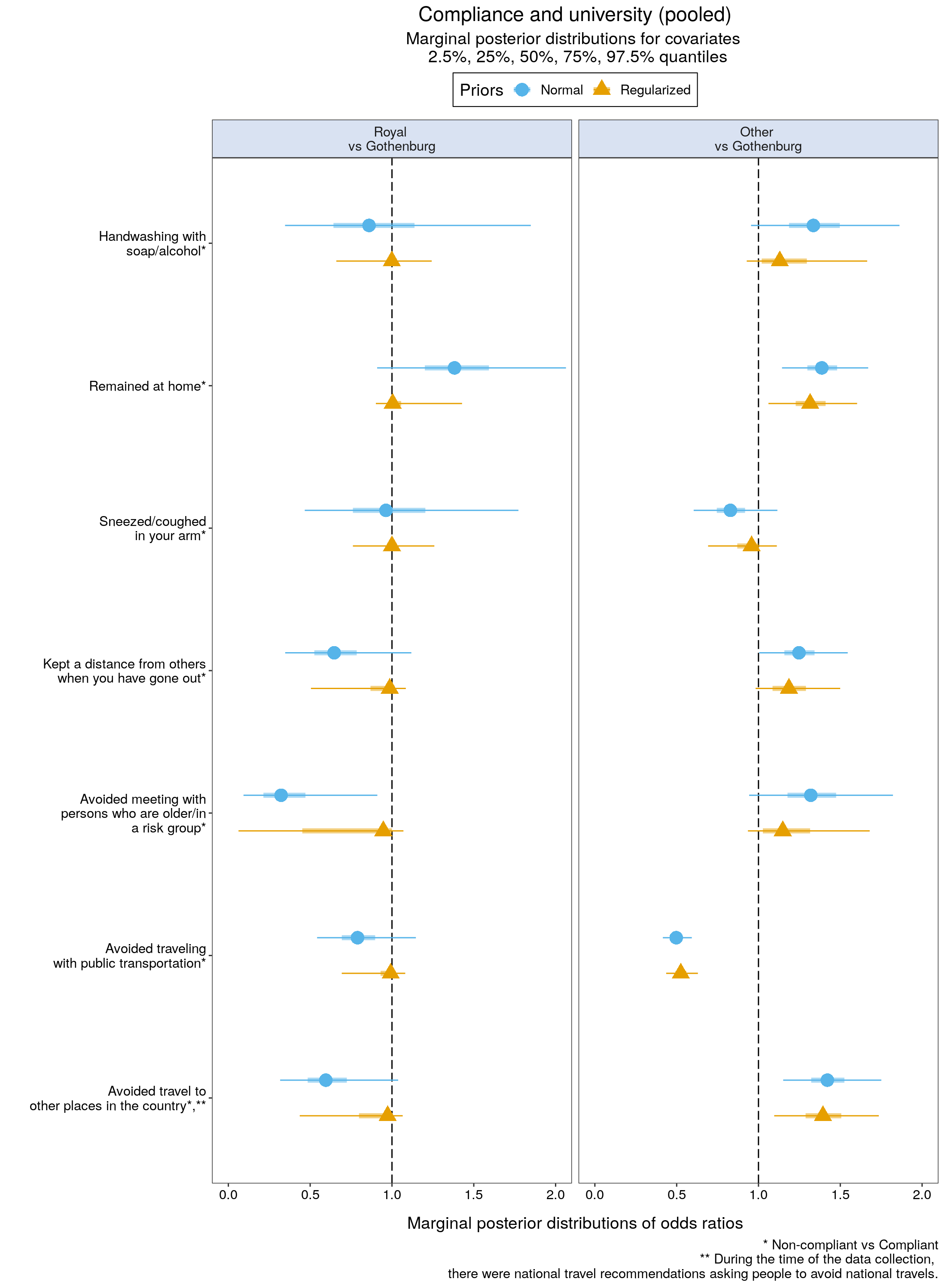


Figure S3 - Marginal posterior distributions using standard normal priors and regularizing priors – Recommendation compliance and university (pooled).
