# Supplementary material for: Compliance with recommendations limiting COVID-19 contagion among university students in Sweden: associations with self-reported symptoms, mental health and academic self-efficacy
Source: Scand J Public Health. 2021 Jul 2;50(1):70–84. doi: 10.1177/14034948211027824 (PMC8808007; doi:10.1177/14034948211027824)
Supplement: sj-docx-2-sjp-10.1177_14034948211027824 – Supplemental material for Compliance with recommendations limiting COVID-19 contagion among university students in Sweden: associations with self-reported symptoms, mental health and academic self-efficacy [file sj-docx-2-sjp-10.1177_14034948211027824.docx]

# Supplementary tables

Table S1 –Self-reported symptoms of contagion and recommendation compliance – Contingency table

| **Self-reported symptoms vs recommendation compliance** | | | | | |
| --- | --- | --- | --- | --- | --- |
|  | **No symptoms** | **Mild symptoms** | **Moderate symptoms** | **Severe symptoms** | **Not relevant/Do not know** |
| **Handwashing with soap/alcohol** | | | | | |
| **Compliance** | 1948 (96.6%) | 893 (96.1%) | 392 (94%) | 62 (95.4%) | 562 (94.3%) |
| **Non-compliance** | 69 (3.4%) | 36 (3.9%) | 25 (6%) | 3 (4.6%) | 34 (5.7%) |
| **Remained at home** | | | | | |
| **Compliance** | 1675 (83%) | 740 (79.8%) | 340 (81.5%) | 56 (87.5%) | 472 (79.2%) |
| **Non-compliance** | 342 (17%) | 187 (20.2%) | 77 (18.5%) | 8 (12.5%) | 124 (20.8%) |
| **Sneezed/coughed in your sleeve** | | | | | |
| **Compliance** | 1873 (93.4%) | 878 (94.6%) | 393 (94.2%) | 61 (93.8%) | 561 (94.3%) |
| **Non-compliance** | 133 (6.6%) | 50 (5.4%) | 24 (5.8%) | 4 (6.2%) | 34 (5.7%) |
| **Kept a distance from others when you have gone out** | | | | | |
| **Compliance** | 1803 (89.4%) | 795 (85.7%) | 363 (87.3%) | 59 (90.8%) | 503 (84.4%) |
| **Non-compliance** | 214 (10.6%) | 133 (14.3%) | 53 (12.7%) | 6 (9.2%) | 93 (15.6%) |
| **Avoided meeting with persons who are older/in a risk group** | | | | | |
| **Compliance** | 1932 (95.9%) | 896 (96.7%) | 396 (95%) | 63 (96.9%) | 561 (94.1%) |
| **Non-compliance** | 82 (4.1%) | 31 (3.3%) | 21 (5%) | 2 (3.1%) | 35 (5.9%) |
| **Avoided traveling with public transportation** | | | | | |
| **Compliance** | 1488 (74%) | 630 (68.2%) | 271 (65%) | 46 (70.8%) | 381 (63.9%) |
| **Non-compliance** | 524 (26%) | 294 (31.8%) | 146 (35%) | 19 (29.2%) | 215 (36.1%) |
| **Avoided travel to other places in the country** | | | | | |
| **Compliance** | 1751 (87.3%) | 794 (85.8%) | 366 (87.8%) | 60 (92.3%) | 511 (86%) |
| **Non-compliance** | 254 (12.7%) | 131 (14.2%) | 51 (12.2%) | 5 (7.7%) | 83 (14%) |

Table S2 –Self-reported symptoms of contagion and recommendation compliance – analytical results

|  | **Bayesian marginal posterior distribution** | | | | **Maximum likelihood estimates and null hypothesis testing** | |
| --- | --- | --- | --- | --- | --- | --- |
|  | **Normal priors** | | **Regularizing priors** | |  |  |
|  | **Median (2.5%; 97.5%)** | **OR > 1** | **Median (2.5%; 97.5%)** | **OR > 1** | **Estimate (95% CI)** | **p-value** |
| **Mild vs No symptoms** | | | | | | |
| **Age** | 0.98 (0.97; 0.99) | 1.0% | 0.98 (0.97; 0.99) | 2.6% | 0.99 (0.97; 1) | 0.073 |
| **Man vs Woman** | 0.94 (0.79; 1.00) | 24.2% | 1.00 (0.90; 1.01) | 44.5% | 0.94 (0.79; 1.13) | 0.521 |
| **Other vs Woman** | 1.23 (0.61; 1.54) | 72.7% | 1.00 (0.88; 1.01) | 52.8% | 1.28 (0.63; 2.63) | 0.496 |
| **Handwashing with soap/alcohol*** | 1.10 (0.72; 1.26) | 67.3% | 1.00 (0.86; 1.01) | 47.9% | 1.12 (0.74; 1.71) | 0.584 |
| **Remained at home*** | 1.12 (0.91; 1.20) | 86.0% | 1.01 (0.96; 1.05) | 68.1% | 1.12 (0.91; 1.38) | 0.278 |
| **Sneezed/coughed in your sleeve*** | 0.86 (0.61; 0.97) | 19.2% | 1.00 (0.83; 1.01) | 43.3% | 0.86 (0.61; 1.2) | 0.373 |
| **Kept a distance from others when you have gone out*** | 1.29 (1.02; 1.40) | 98.3% | 1.01 (0.97; 1.08) | 71.8% | 1.31 (1.03; 1.66) | 0.028 |
| **Avoided meeting with persons who are older/in a risk group*** | 0.78 (0.51; 0.90) | 11.6% | 1.00 (0.71; 1.00) | 37.2% | 0.78 (0.51; 1.19) | 0.247 |
| **Avoided traveling with public transportation*** | 1.19 (0.99; 1.27) | 97.0% | 1.01 (0.97; 1.07) | 73.0% | 1.20 (1.00; 1.45) | 0.045 |
| **Avoided travel to other places in the country*** | 1.05 (0.83; 1.14) | 65.9% | 1.00 (0.95; 1.03) | 61.6% | 1.05 (0.83; 1.33) | 0.678 |
| **Moderate vs No symptoms** | | | | | | |
| **Age** | 0.99 (0.97; 0.99) | 7.4% | 0.99 (0.97; 1.00) | 17.5% | 0.99 (0.97; 1.01) | 0.429 |
| **Man vs Woman** | 0.87 (0.68; 0.95) | 13.7% | 1.00 (0.87; 1.00) | 42.5% | 0.88 (0.68; 1.12) | 0.302 |
| **Other vs Woman** | 0.71 (0.24; 0.99) | 24.7% | 1.00 (0.73; 1.01) | 46.2% | 0.69 (0.20; 2.34) | 0.553 |
| **Handwashing with soap/alcohol*** | 1.75 (1.06; 2.05) | 98.6% | 1.00 (0.94; 1.06) | 64.4% | 1.83 (1.13; 2.95) | 0.014 |
| **Remained at home*** | 0.95 (0.71; 1.04) | 35.3% | 1.00 (0.88; 1.01) | 47.0% | 0.95 (0.71; 1.26) | 0.718 |
| **Sneezed/coughed in your sleeve*** | 0.89 (0.56; 1.04) | 30.9% | 1.00 (0.87; 1.01) | 48.8% | 0.89 (0.56; 1.40) | 0.604 |
| **Kept a distance from others when you have gone out*** | 1.08 (0.78; 1.21) | 68.4% | 1.00 (0.90; 1.01) | 50.5% | 1.11 (0.79; 1.54) | 0.557 |
| **Avoided meeting with persons who are older/in a risk group*** | 1.21 (0.72; 1.43) | 77.8% | 1.00 (0.91; 1.02) | 55.3% | 1.23 (0.75; 2.04) | 0.412 |
| **Avoided traveling with public transportation*** | 1.47 (1.16; 1.60) | 99.9% | 1.10 (0.98; 1.27) | 83.2% | 1.50 (1.18; 1.91) | 0.001 |
| **Avoided travel to other places in the country*** | 0.84 (0.60; 0.94) | 14.8% | 1.00 (0.84; 1.01) | 44.4% | 0.84 (0.60; 1.18) | 0.322 |
| **Severe vs No symptoms** | | | | | | |
| **Age** | 0.95 (0.91; 0.96) | 0.5% | 0.97 (0.92; 0.99) | 7.2% | 0.98 (0.94; 1.03) | 0.505 |
| **Man vs Woman** | 1.57 (0.93; 1.87) | 95.2% | 1.00 (0.93; 1.06) | 63.3% | 1.76 (1.03; 2.98) | 0.038 |
| **Other vs Woman** | 1.20 (0.24; 1.97) | 59.4% | 1.00 (0.76; 1.02) | 50.6% | 1.82 (0.24; 13.94) | 0.564 |
| **Handwashing with soap/alcohol*** | 0.80 (0.22; 1.18) | 35.1% | 1.00 (0.70; 1.01) | 48.0% | 0.82 (0.19; 3.45) | 0.782 |
| **Remained at home*** | 0.68 (0.32; 0.86) | 13.0% | 1.00 (0.63; 1.01) | 41.2% | 0.68 (0.31; 1.47) | 0.323 |
| **Sneezed/coughed in your sleeve*** | 0.94 (0.34; 1.29) | 45.0% | 1.00 (0.79; 1.01) | 50.3% | 0.97 (0.34; 2.74) | 0.949 |
| **Kept a distance from others when you have gone out*** | 0.77 (0.32; 1.01) | 25.5% | 1.00 (0.69; 1.01) | 44.5% | 0.82 (0.34; 1.96) | 0.655 |
| **Avoided meeting with persons who are older/in a risk group*** | 0.81 (0.23; 1.18) | 34.9% | 1.00 (0.70; 1.01) | 47.9% | 0.81 (0.19; 3.41) | 0.772 |
| **Avoided traveling with public transportation*** | 1.24 (0.70; 1.50) | 77.4% | 1.00 (0.85; 1.01) | 50.1% | 1.42 (0.8; 2.53) | 0.226 |
| **Avoided travel to other places in the country*** | 0.46 (0.18; 0.62) | 3.0% | 1.00 (0.41; 1.00) | 37.3% | 0.42 (0.15; 1.20) | 0.106 |
| **Not relevant/do not know vs No symptoms** | | | | | | |
| **Age** | 1.01 (1.00; 1.02) | 92.0% | 1.01 (1.00; 1.01) | 85.6% | 1.02 (1.00; 1.04) | 0.046 |
| **Man vs Woman** | 0.95 (0.77; 1.03) | 33.2% | 1.00 (0.9; 1.02) | 50.3% | 0.96 (0.78; 1.19) | 0.719 |
| **Other vs Woman** | 1.38 (0.63; 1.77) | 79.7% | 1.00 (0.85; 1.03) | 56.3% | 1.48 (0.67; 3.28) | 0.33 |
| **Handwashing with soap/alcohol*** | 1.59 (1.04; 1.84) | 98.4% | 1.02 (0.94; 1.13) | 69.7% | 1.65 (1.07; 2.54) | 0.023 |
| **Remained at home*** | 1.09 (0.85; 1.18) | 75.4% | 1.00 (0.93; 1.04) | 62.1% | 1.09 (0.86; 1.38) | 0.491 |
| **Sneezed/coughed in your sleeve*** | 0.83 (0.55; 0.94) | 16.5% | 1.00 (0.81; 1.01) | 45.0% | 0.82 (0.55; 1.22) | 0.321 |
| **Kept a distance from others when you have gone out*** | 1.41 (1.07; 1.54) | 99.4% | 1.06 (0.97; 1.22) | 81.2% | 1.43 (1.09; 1.88) | 0.01 |
| **Avoided meeting with persons who are older/in a risk group*** | 1.33 (0.88; 1.52) | 90.9% | 1.01 (0.93; 1.10) | 67.8% | 1.34 (0.88; 2.04) | 0.169 |
| **Avoided traveling with public transportation*** | 1.53 (1.25; 1.64) | > 99.9% | 1.37 (1.02; 1.48) | 99.0% | 1.56 (1.27; 1.92) | < 0.001 |
| **Avoided travel to other places in the country*** | 0.92 (0.69; 1.02) | 28.7% | 1.00 (0.87; 1.01) | 49.0% | 0.93 (0.70; 1.23) | 0.601 |
| * Non-compliant vs compliant | | | | | | |

Table S3 - Self-reported symptoms of contagion and effects on mental health – Contingency table

| **Self-reported mental health effects vs self-reported symptoms** | | | | |
| --- | --- | --- | --- | --- |
|  | **No effect** | **Mental health has been worse** | **Mental health has been better** | **Mental health has been both better and worse** |
| **Self-reported symptoms** | | | | |
| **No symptoms** | 426 (61.6%) | 797 (48%) | 160 (54.6%) | 601 (52%) |
| **Mild symptoms** | 139 (20.1%) | 397 (23.9%) | 62 (21.2%) | 245 (21.2%) |
| **Moderate symptoms** | 35 (5.1%) | 185 (11.1%) | 27 (9.2%) | 95 (8.2%) |
| **Severe symptoms** | 2 (0.3%) | 33 (2%) | 5 (1.7%) | 8 (0.7%) |
| **Not relevant/Do not know** | 89 (12.9%) | 249 (15%) | 39 (13.3%) | 207 (17.9%) |

Table S4 - Self-reported symptoms of contagion and effects on mental health – Analytical results

|  | **Bayesian marginal posterior distribution** | | | | **Maximum likelihood estimates and null hypothesis testing** | |
| --- | --- | --- | --- | --- | --- | --- |
|  | **Normal priors** | | **Regularizing priors** | |  |  |
|  | **Median (2.5%; 97.5%)** | **OR > 1** | **Median (2.5%; 97.5%)** | **OR > 1** | **Estimate (95% CI)** | **p-value** |
| **My mental health has been worse vs No effect** | | | | | | |
| Age | 0.92 (0.91; 0.93) | < 0.01% | 0.93 (0.91; 0.93) | < 0.01% | 0.91 (0.9; 0.93) | < 0.001 |
| **Man vs Woman** | 0.69 (0.57; 0.74) | < 0.01% | 0.79 (0.65; 0.85) | 0.8% | 0.67 (0.55; 0.81) | < 0.001 |
| **Other vs Woman** | 1.18 (0.54; 1.54) | 65.7% | 1.00 (0.69; 1.09) | 52.9% | 1.25 (0.49; 3.19) | 0.645 |
| Mild vs No symptoms | 1.46 (1.17; 1.58) | > 99.9% | 1.26 (1.03; 1.35) | 99.3% | 1.47 (1.17; 1.85) | 0.001 |
| Moderate vs No symptoms | 2.62 (1.83; 2.97) | > 99.9% | 1.77 (1.32; 1.98) | > 99.9% | 2.79 (1.9; 4.11) | < 0.001 |
| Severe vs No symptoms | 4.08 (1.91; 5.42) | > 99.9% | 2.71 (1.22; 3.48) | 99.6% | 9.54 (2.26; 40.21) | 0.002 |
| Not relevant/do not know vs No symptoms | 1.62 (1.23; 1.78) | > 99.9% | 1.31 (0.99; 1.45) | 96.6% | 1.66 (1.25; 2.19) | < 0.001 |
| **My mental health has been better vs No effect** | | | | | | |
| Age | 0.95 (0.93; 0.96) | < 0.01% | 0.96 (0.94; 0.97) | 0.3% | 0.94 (0.92; 0.97) | < 0.001 |
| **Man vs Woman** | 0.71 (0.52; 0.78) | 0.9% | 0.98 (0.66; 1.00) | 27.7% | 0.68 (0.50; 0.92) | 0.012 |
| **Other vs Woman** | 1.23 (0.41; 1.77) | 65.2% | 1.00 (0.80; 1.02) | 52.1% | 1.45 (0.40; 5.24) | 0.567 |
| Mild vs No symptoms | 1.11 (0.78; 1.25) | 71.8% | 1.00 (0.85; 1.01) | 46.1% | 1.12 (0.79; 1.6) | 0.516 |
| Moderate vs No symptoms | 1.84 (1.09; 2.19) | 98.8% | 1.01 (0.91; 1.06) | 62.7% | 2.03 (1.19; 3.48) | 0.009 |
| Severe vs No symptoms | 2.4 (0.78; 3.49) | 93.8% | 1.00 (0.87; 1.04) | 59.2% | 6.96 (1.33; 36.35) | 0.021 |
| Not relevant/do not know vs No symptoms | 1.18 (0.78; 1.36) | 78.5% | 1.00 (0.82; 1.01) | 48.5% | 1.23 (0.80; 1.88) | 0.346 |
| **My mental health has been both better and worse vs No effect** | | | | | | |
| Age | 0.93 (0.92; 0.94) | < 0.01% | 0.94 (0.92; 0.94) | < 0.01% | 0.93 (0.91; 0.94) | < 0.001 |
| **Man vs Woman** | 0.48 (0.39; 0.52) | < 0.01% | 0.55 (0.44; 0.59) | < 0.01% | 0.47 (0.38; 0.58) | < 0.001 |
| **Other vs Woman** | 1.19 (0.54; 1.56) | 66.6% | 1.00 (0.74; 1.05) | 52.3% | 1.27 (0.48; 3.33) | 0.628 |
| Mild vs No symptoms | 1.19 (0.94; 1.3) | 92.1% | 1.01 (0.90; 1.06) | 61.0% | 1.19 (0.93; 1.53) | 0.158 |
| Moderate vs No symptoms | 1.78 (1.21; 2.04) | 99.9% | 1.09 (0.93; 1.26) | 80.8% | 1.90 (1.26; 2.87) | 0.002 |
| Severe vs No symptoms | 1.33 (0.51; 1.86) | 72.2% | 1.00 (0.61; 1.04) | 47.4% | 3.19 (0.67; 15.20) | 0.145 |
| Not relevant/do not know vs No symptoms | 1.76 (1.33; 1.93) | >99.9% | 1.41 (1.01; 1.56) | 98.6% | 1.80 (1.35; 2.40) | < 0.001 |
| * Non-compliant vs Compliant | | | | | | |

Table S5 - Self-reported symptoms of contagion and change in academic self-efficacy – Contingency table

| **Self-reported change in academic self-efficacy vs self-reported symptoms** | | | | | |
| --- | --- | --- | --- | --- | --- |
|  | **No change** | **Studies have been going worse** | **Studies have been going better** | **Studies have been going both better and worse** | **I am not studying at this time** |
| **Self-reported symptoms** | | | | | |
| **No symptoms** | 297 (63.1%) | 800 (49.3%) | 142 (55.9%) | 662 (51.4%) | 40 (45.5%) |
| **Mild symptoms** | 87 (18.5%) | 382 (23.5%) | 53 (20.9%) | 293 (22.7%) | 17 (19.3%) |
| **Moderate symptoms** | 20 (4.2%) | 174 (10.7%) | 26 (10.2%) | 103 (8%) | 11 (12.5%) |
| **Severe symptoms** | 1 (0.2%) | 30 (1.8%) | 1 (0.4%) | 8 (0.6%) | 6 (6.8%) |
| **Not relevant/Do not know** | 66 (14%) | 238 (14.7%) | 32 (12.6%) | 223 (17.3%) | 14 (15.9%) |

Table S6 - Self-reported symptoms of contagion and change in academic self-efficacy – Analytical results

|  | **Bayesian marginal posterior distribution** | | | | **Maximum likelihood estimates and null hypothesis testing** | |
| --- | --- | --- | --- | --- | --- | --- |
|  | **Normal priors** | | **Regularizing priors** | |  |  |
|  | **Median (2.5%; 97.5%)** | **OR > 1** | **Median (2.5%; 97.5%)** | **OR > 1** | **Estimate (95% CI)** | **p-value** |
| **My studies have been going worse vs No change** | | | | | | |
| Age | 0.91 (0.90; 0.92) | < 0.01% | 0.92 (0.90; 0.92) | < 0.01% | 0.90 (0.88; 0.92) | < 0.001 |
| **Man vs Woman** | 0.97 (0.77; 1.05) | 40% | 1.06 (0.95; 1.13) | 81.2% | 0.94 (0.74; 1.18) | 0.586 |
| **Other vs Woman** | 2.42 (1.03; 3.31) | 98% | 1.06 (0.87; 1.31) | 72.4% | 3.76 (0.87; 16.24) | 0.077 |
| Mild vs No symptoms | 1.54 (1.20; 1.69) | > 99.9% | 1.13 (0.98; 1.23) | 92.1% | 1.57 (1.20; 2.06) | 0.001 |
| Moderate vs No symptoms | 2.81 (1.86; 3.28) | >99.9% | 1.45 (1.05; 1.61) | 99.4% | 3.39 (2.06; 5.57) | < 0.001 |
| Severe vs No symptoms | 3.03 (1.39; 4.02) | 99.7% | 2.6 (1.00; 3.54) | 96.8% | 11.43 (1.54; 84.79) | 0.017 |
| Not relevant/do not know vs No symptoms | 1.38 (1.03; 1.52) | 98.4% | 1.02 (0.91; 1.10) | 68.7% | 1.42 (1.04; 1.94) | 0.027 |
| **My studies have been going better vs No change** | | | | | | |
| Age | 0.97 (0.95; 0.98) | 1.7% | 0.99 (0.96; 1.00) | 10.5% | 0.96 (0.94; 0.99) | 0.007 |
| **Man vs Woman** | 0.71 (0.50; 0.80) | 2.9% | 1.00 (0.72; 1.00) | 35.3% | 0.69 (0.48; 0.99) | 0.041 |
| **Other vs Woman** | 1.07 (0.27; 1.65) | 53.9% | 1.00 (0.700; 1.01) | 46.6% | 1.82 (0.25; 13.06) | 0.552 |
| Mild vs No symptoms | 1.20 (0.81; 1.37) | 81.8% | 1.00 (0.87; 1.01) | 47.9% | 1.23 (0.82; 1.83) | 0.314 |
| Moderate vs No symptoms | 2.20 (1.25; 2.67) | 99.7% | 1.00 (0.92; 1.03) | 59.7% | 2.74 (1.46; 5.15) | 0.002 |
| Severe vs No symptoms | 0.69 (0.16; 1.10) | 29.6% | 1.00 (0.67; 1.01) | 48.7% | 2.17 (0.13; 35.03) | 0.585 |
| Not relevant/do not know vs No symptoms | 0.95 (0.59; 1.11) | 41.4% | 1.00 (0.68; 1.00) | 36.5% | 0.99 (0.61; 1.59) | 0.951 |
| **My studies have been going both better and worse (in differing ways) vs No change** | | | | | | |
| Age | 0.91 (0.89; 0.92) | < 0.01% | 0.91 (0.90; 0.92) | < 0.01% | 0.90 (0.88; 0.92) | < 0.001 |
| **Man vs Woman** | 0.81 (0.64; 0.88) | 4.0% | 0.98 (0.80; 1.00) | 25.8% | 0.78 (0.61; 0.99) | 0.043 |
| **Other vs Woman** | 1.98 (0.82; 2.71) | 93.2% | 1.00 (0.84; 1.05) | 56.9% | 3.12 (0.70; 13.82) | 0.134 |
| Mild vs No symptoms | 1.42 (1.09; 1.56) | 99.5% | 1.01 (0.94; 1.07) | 67.9% | 1.44 (1.09; 1.91) | 0.011 |
| Moderate vs No symptoms | 2.03 (1.32; 2.37) | >99.9% | 1.01 (0.90; 1.05) | 61.7% | 2.44 (1.46; 4.08) | 0.001 |
| Severe vs No symptoms | 1.01 (0.39; 1.41) | 51% | 1.00 (0.55; 1.02) | 45.8% | 3.80 (0.47; 30.72) | 0.211 |
| Not relevant/do not know vs No symptoms | 1.55 (1.15; 1.72) | 99.8% | 1.09 (0.97; 1.23) | 84.3% | 1.60 (1.17; 2.19) | 0.004 |
| **I am not studying at this time vs No change** | | | | | | |
| Age | 1.00 (0.96; 1.01) | 49.4% | 1.00 (0.98; 1.01) | 55.7% | 1.01 (0.97; 1.05) | 0.639 |
| **Man vs Woman** | 0.73 (0.43; 0.87) | 11.5% | 1.00 (0.70; 1.01) | 41.9% | 0.72 (0.42; 1.24) | 0.231 |
| **Other vs Woman** | 0.62 (0.11; 1.10) | 28.4% | 1.00 (0.18; 1.01) | 43.1% | NA^a^ | NA^a^ |
| Mild vs No symptoms | 1.2 (0.65; 1.47) | 72.3% | 1.00 (0.78; 1.01) | 47.1% | 1.44 (0.76; 2.7) | 0.261 |
| Moderate vs No symptoms | 2.91 (1.34; 3.76) | 99.6% | 1.01 (0.91; 1.12) | 65.1% | 4.48 (1.98; 10.14) | < 0.001 |
| Severe vs No symptoms | 7.34 (2.35; 10.69) | 99.9% | 7.05 (0.99; 11.95) | 90.8% | 48.5 (5.67; 414.83) | < 0.001 |
| Not relevant/do not know vs No symptoms | 1.18 (0.60; 1.48) | 69.6% | 1.00 (0.77; 1.01) | 47.3% | 1.43 (0.71; 2.88) | 0.322 |
| * Non-compliant vs Compliant ^a^ Not estimable in the MLE model due to zero entries | | | | | | |

Table S7 – Recommendation compliance and gender – Contingency table

| **Recommendation compliance and gender** | | | |
| --- | --- | --- | --- |
|  | **Gender - Female** | **Gender - Male** | **Gender - Other** |
| **Handwashing with soap/alcohol** | | | |
| **Compliance** | 2850 (97.4%) | 1031 (91.6%) | 46 (95.8%) |
| **Non-compliance** | 77 (2.6%) | 95 (8.4%) | 2 (4.2%) |
| **Remained at home** | | | |
| **Compliance** | 2390 (81.7%) | 902 (80.2%) | 38 (79.2%) |
| **Non-compliance** | 537 (18.3%) | 223 (19.8%) | 10 (20.8%) |
| **Sneezed/coughed in your sleeve** | | | |
| **Compliance** | 2783 (95.4%) | 1008 (89.8%) | 42 (87.5%) |
| **Non-compliance** | 135 (4.6%) | 115 (10.2%) | 6 (12.5%) |
| **Kept a distance from others when you have gone out** | | | |
| **Compliance** | 2626 (89.8%) | 905 (80.4%) | 42 (87.5%) |
| **Non-compliance** | 299 (10.2%) | 221 (19.6%) | 6 (12.5%) |
| **Avoided meeting with persons who are older/in a risk group** | | | |
| **Compliance** | 2820 (96.4%) | 1049 (93.4%) | 44 (91.7%) |
| **Non-compliance** | 106 (3.6%) | 74 (6.6%) | 4 (8.3%) |
| **Avoided traveling with public transportation** | | | |
| **Compliance** | 2060 (70.5%) | 757 (67.5%) | 36 (75%) |
| **Non-compliance** | 863 (29.5%) | 365 (32.5%) | 12 (25%) |
| **Avoided travel to other places in the country** | | | |
| **Compliance** | 2575 (88.3%) | 917 (82%) | 43 (89.6%) |
| **Non-compliance** | 342 (11.7%) | 201 (18%) | 5 (10.4%) |

Table S8 - Recommendation compliance and gender – Analytical results

|  | **Bayesian marginal posterior distribution** | | | | **Maximum likelihood estimates and null hypothesis testing** | |
| --- | --- | --- | --- | --- | --- | --- |
|  | **Normal priors** | | **Regularizing priors** | |  |  |
|  | **Median (2.5%; 97.5%)** | **OR > 1** | **Median (2.5%; 97.5%)** | **OR > 1** | **Estimate (95% CI)** | **p-value** |
| **Man vs Woman** | | | | | | |
| Handwashing with soap/alcohol* | 2.91 (2.12; 3.24) | >99.9% | 2.86 (2.08; 3.19) | >99.9% | 2.99 (2.17; 4.11) | < 0.01 |
| Remained at home* | 0.97 (0.81; 1.04) | 38.1% | 0.99 (0.85; 1.02) | 42.5% | 0.97 (0.81; 1.17) | 0.781 |
| Sneezed/coughed in your sleeve* | 2.03 (1.55; 2.22) | >99.9% | 1.95 (1.47; 2.14) | >99.9% | 2.07 (1.58; 2.7) | < 0.01 |
| Kept a distance from others when you have gone out* | 1.97 (1.62; 2.10) | >99.9% | 1.94 (1.59; 2.08) | >99.)% | 1.98 (1.63; 2.42) | < 0.01 |
| Avoided meeting with persons who are older/in a risk group* | 1.38 (0.99; 1.54) | 97.3% | 1.20 (0.95; 1.37) | 90.1% | 1.39 (1.01; 1.93) | 0.045 |
| Avoided traveling with public transportation* | 0.97 (0.83; 1.03) | 37.6% | 1.00 (0.87; 1.02) | 44.7% | 0.97 (0.83; 1.15) | 0.757 |
| Avoided travel to other places in the country* | 1.42 (1.16; 1.52) | >99.9% | 1.38 (1.10; 1.48) | 99.9% | 1.42 (1.16; 1.74) | 0.001 |
| **Other vs Woman** | | | | | | |
| Handwashing with soap/alcohol* | 1.16 (0.30; 1.72) | 59.6% | 1.00 (0.81; 1.01) | 51.8% | 1.46 (0.35; 6.19) | 0.605 |
| Remained at home* | 1.12 (0.55; 1.41) | 62.2% | 1.00 (0.84; 1.01) | 51.1% | 1.26 (0.60; 2.62) | 0.537 |
| Sneezed/coughed in your sleeve* | 2.09 (0.83; 2.77) | 94.7% | 1.00 (0.90; 1.04) | 59.3% | 2.72 (1.13; 6.59) | 0.026 |
| Kept a distance from others when you have gone out* | 1.07 (0.44; 1.40) | 56.6% | 1.00 (0.83; 1.01) | 51.3% | 1.19 (0.49; 2.88) | 0.699 |
| Avoided meeting with persons who are older/in a risk group* | 1.71 (0.57; 2.36) | 84.3% | 1.00 (0.86; 1.02) | 55.3% | 2.20 (0.76; 6.38) | 0.148 |
| Avoided traveling with public transportation* | 0.70 (0.36; 0.87) | 13.1% | 1.00 (0.70; 1.01) | 43.1% | 0.74 (0.37; 1.47) | 0.386 |
| Avoided travel to other places in the country* | 0.80 (0.32; 1.07) | 30.3% | 1.00 (0.75; 1.01) | 47.2% | 0.82 (0.31; 2.15) | 0.69 |
| * Non-compliant vs Compliant | | | | | | |

Table S9 - Recommendation compliance and age (pooled) – Contingency table

| **Recommendation compliance and age (pooled)** | | | |
| --- | --- | --- | --- |
|  | **16-25** | **26-35** | **36+** |
| **Handwashing with soap/alcohol** | | | |
| **Compliance** | 2053 (95.5%) | 1313 (96%) | 524 (95.8%) |
| **Non-compliance** | 97 (4.5%) | 55 (4%) | 23 (4.2%) |
| **Remained at home** | | | |
| **Compliance** | 1712 (79.6%) | 1141 (83.5%) | 447 (81.9%) |
| **Non-compliance** | 440 (20.4%) | 225 (16.5%) | 99 (18.1%) |
| **Sneezed/coughed in your sleeve** | | | |
| **Compliance** | 2032 (94.8%) | 1270 (93%) | 497 (91.2%) |
| **Non-compliance** | 112 (5.2%) | 95 (7%) | 48 (8.8%) |
| **Kept a distance from others when you have gone out** | | | |
| **Compliance** | 1813 (84.2%) | 1220 (89.4%) | 507 (92.9%) |
| **Non-compliance** | 339 (15.8%) | 145 (10.6%) | 39 (7.1%) |
| **Avoided meeting with persons who are older/in a risk group** | | | |
| **Compliance** | 2062 (95.9%) | 1302 (95.2%) | 514 (94.5%) |
| **Non-compliance** | 89 (4.1%) | 65 (4.8%) | 30 (5.5%) |
| **Avoided traveling with public transportation** | | | |
| **Compliance** | 1410 (65.6%) | 964 (70.8%) | 452 (82.9%) |
| **Non-compliance** | 741 (34.4%) | 397 (29.2%) | 93 (17.1%) |
| **Avoided travel to other places in the country** | | | |
| **Compliance** | 1786 (83.3%) | 1225 (89.9%) | 495 (91.7%) |
| **Non-compliance** | 359 (16.7%) | 137 (10.1%) | 45 (8.3%) |

Table S10 - Recommendation compliance and age (pooled) – Analytical results

|  | **Bayesian marginal posterior distribution** | | | | **Maximum likelihood estimates and null hypothesis testing** | |
| --- | --- | --- | --- | --- | --- | --- |
|  | **Normal priors** | | **Regularizing priors** | |  |  |
|  | **Median (2.5%; 97.5%)** | **OR > 1** | **Median (2.5%; 97.5%)** | **OR > 1** | **Estimate (95% CI)** | **p-value** |
| **26-35 vs 16-25** | | | | | | |
| Handwashing with soap/alcohol* | 0.93 (0.66; 1.04) | 32.6% | 0.99 (0.77; 1.02) | 42.0% | 0.92 (0.65; 1.31) | 0.657 |
| Remained at home* | 0.86 (0.71; 0.92) | 5.3% | 0.90 (0.73; 0.97) | 10.6% | 0.86 (0.72; 1.04) | 0.117 |
| Sneezed/coughed in your sleeve* | 1.39 (1.04; 1.54) | 98.9% | 1.16 (0.96; 1.32) | 88.1% | 1.41 (1.06; 1.88) | 0.018 |
| Kept a distance from others when you have gone out* | 0.68 (0.55; 0.74) | < 0.01% | 0.73 (0.58; 0.79) | 0.6% | 0.68 (0.55; 0.84) | < 0.01 |
| Avoided meeting with persons who are older/in a risk group* | 1.32 (0.94; 1.48) | 94.7% | 1.05 (0.91; 1.18) | 75.2% | 1.35 (0.96; 1.89) | 0.083 |
| Avoided traveling with public transportation* | 0.90 (0.77; 0.95) | 9.0% | 0.95 (0.80; 0.99) | 17.6% | 0.90 (0.77; 1.05) | 0.183 |
| Avoided travel to other places in the country* | 0.59 (0.47; 0.63) | < 0.01% | 0.61 (0.49; 0.66) | < 0.01% | 0.59 (0.47; 0.73) | < 0.01 |
| **36+ vs 16-25** | | | | | | |
| Handwashing with soap/alcohol* | 1.03 (0.64; 1.21) | 55.0% | 1.01 (0.74; 1.1) | 55.7% | 1.04 (0.64; 1.67) | 0.875 |
| Remained at home* | 1.23 (0.95; 1.34) | 94.1% | 1.14 (0.94; 1.26) | 87.4% | 1.25 (0.96; 1.61) | 0.093 |
| Sneezed/coughed in your sleeve* | 1.73 (1.19; 1.96) | 99.8% | 1.47 (0.99; 1.7) | 96.9% | 1.78 (1.23; 2.57) | 0.002 |
| Kept a distance from others when you have gone out* | 0.47 (0.33; 0.53) | < 0.01% | 0.51 (0.35; 0.58) | < 0.01% | 0.46 (0.32; 0.66) | < 0.001 |
| Avoided meeting with persons who are older/in a risk group* | 1.73 (1.10; 2.01) | 99.1% | 1.37 (0.95; 1.64) | 92.2% | 1.81 (1.16; 2.82) | 0.009 |
| Avoided traveling with public transportation* | 0.43 (0.33; 0.46) | < 0.01% | 0.45 (0.34; 0.49) | < 0.01% | 0.42 (0.33; 0.55) | < 0.001 |
| Avoided travel to other places in the country* | 0.52 (0.36; 0.58) | < 0.01% | 0.56 (0.39; 0.63) | 0.1% | 0.51 (0.36; 0.72) | < 0.001 |

Table S11 - Recommendation compliance and university (pooled) – Contingency table

| **Recommendation compliance and university (pooled)** | | | |
| --- | --- | --- | --- |
|  | **Gothenburg** | **Royal** | **Other** |
| **Handwashing with soap/alcohol** | | | |
| **Compliance** | 2825 (96%) | 147 (96.7%) | 959 (94.8%) |
| **Non-compliance** | 117 (4%) | 5 (3.3%) | 53 (5.2%) |
| **Remained at home** | | | |
| **Compliance** | 2418 (82.2%) | 120 (78.9%) | 795 (78.8%) |
| **Non-compliance** | 524 (17.8%) | 32 (21.1%) | 214 (21.2%) |
| **Sneezed/coughed in your sleeve** | | | |
| **Compliance** | 2744 (93.5%) | 142 (94%) | 949 (94.1%) |
| **Non-compliance** | 190 (6.5%) | 9 (6%) | 59 (5.9%) |
| **Kept a distance from others when you have gone out** | | | |
| **Compliance** | 2578 (87.6%) | 140 (92.1%) | 860 (85.1%) |
| **Non-compliance** | 364 (12.4%) | 12 (7.9%) | 150 (14.9%) |
| **Avoided meeting with persons who are older/in a risk group** | | | |
| **Compliance** | 2814 (95.7%) | 151 (99.3%) | 952 (94.4%) |
| **Non-compliance** | 126 (4.3%) | 1 (0.7%) | 57 (5.6%) |
| **Avoided traveling with public transportation** | | | |
| **Compliance** | 1966 (67%) | 110 (72.4%) | 780 (77.4%) |
| **Non-compliance** | 970 (33%) | 42 (27.6%) | 228 (22.6%) |
| **Avoided travel to other places in the country** | | | |
| **Compliance** | 2552 (87.1%) | 141 (92.8%) | 845 (84%) |
| **Non-compliance** | 377 (12.9%) | 11 (7.2%) | 161 (16%) |

Table S12 - Recommendation compliance and university (pooled) – Analytical results

|  | **Bayesian marginal posterior distribution** | | | | **Maximum likelihood estimates and null hypothesis testing** | | |
| --- | --- | --- | --- | --- | --- | --- | --- |
|  | **Normal priors** | | **Regularizing priors** | |  |  | |
|  | **Median (2.5%; 97.5%)** | **OR > 1** | **Median (2.5%; 97.5%)** | **OR > 1** | **Estimate (95% CI)** | **p-value** |  |
| **Royal vs Gothenburg** | | | | | | |  |
| Handwashing with soap/alcohol* | 0.86 (0.35; 1.14) | 36.1% | 1.00 (0.66; 1.01) | 45.6% | 0.90 (0.36; 2.25) | 0.822 |  |
| Remained at home* | 1.38 (0.91; 1.59) | 93.5% | 1.00 (0.90; 1.06) | 62.0% | 1.45 (0.95; 2.21) | 0.083 |  |
| Sneezed/coughed in your sleeve* | 0.96 (0.47; 1.20) | 45.5% | 1.00 (0.76; 1.02) | 49.1% | 1.02 (0.51; 2.05) | 0.945 |  |
| Kept a distance from others when you have gone out* | 0.65 (0.35; 0.78) | 6.0% | 0.99 (0.50; 1.00) | 31.1% | 0.65 (0.36; 1.2) | 0.168 |  |
| Avoided meeting with persons who are older/in a risk group* | 0.32 (0.09; 0.47) | 1.5% | 0.95 (0.06; 1.00) | 25.3% | 0.17 (0.02; 1.23) | 0.079 |  |
| Avoided traveling with public transportation* | 0.79 (0.54; 0.90) | 10.8% | 0.99 (0.69; 1.00) | 34.0% | 0.81 (0.55; 1.19) | 0.282 |  |
| Avoided travel to other places in the country* | 0.60 (0.32; 0.72) | 3.5% | 0.97 (0.44; 1.00) | 27.6% | 0.59 (0.31; 1.12) | 0.107 |  |
| **Other vs Gothenburg** | | | | | | |  |
| Handwashing with soap/alcohol* | 1.34 (0.95; 1.5) | 95.4% | 1.13 (0.93; 1.30) | 83.9% | 1.35 (0.96; 1.90) | 0.082 |  |
| Remained at home* | 1.39 (1.14; 1.48) | >99.9% | 1.32 (1.06; 1.41) | 99.6% | 1.39 (1.15; 1.68) | 0.001 |  |
| Sneezed/coughed in your sleeve* | 0.83 (0.60; 0.92) | 11.2% | 0.96 (0.69; 1.00) | 27.3% | 0.83 (0.61; 1.13) | 0.232 |  |
| Kept a distance from others when you have gone out* | 1.25 (1.00; 1.34) | 97.8% | 1.19 (0.98; 1.29) | 94.2% | 1.25 (1.01; 1.55) | 0.04 |  |
| Avoided meeting with persons who are older/in a risk group* | 1.32 (0.94; 1.47) | 95% | 1.15 (0.94; 1.32) | 85% | 1.32 (0.95; 1.85) | 0.098 |  |
| Avoided traveling with public transportation* | 0.50 (0.42; 0.53) | < 0.01% | 0.53 (0.44; 0.56) | < 0.01% | 0.50 (0.42; 0.59) | < 0.01 |  |
| Avoided travel to other places in the country* | 1.42 (1.15; 1.53) | 99.9% | 1.39 (1.10; 1.51) | 99.7% | 1.43 (1.15; 1.76) | 0.001 |  |
| * Non-compliant vs Compliant | | | | | | |  |
